# Silencing of cZNF292 circular RNA suppresses human glioma tube formation via the Wnt/β-catenin signaling pathway

**DOI:** 10.18632/oncotarget.11523

**Published:** 2016-08-23

**Authors:** Ping Yang, Zhijun Qiu, Yuan Jiang, Lei Dong, Wensheng Yang, Chao Gu, Guang Li, Yu Zhu

**Affiliations:** ^1^ Department of Clinical Laboratory, Tianjin Huanhu Hospital, Tianjin Key Laboratory of Cerebral Vessels and Neural Degeneration, Tianjin, China; ^2^ Department of Neurology, Tianjin Medical University General Hospital, Tianjin Neurological Institute, Tianjin, China; ^3^ Department of Immunology, Tianjin Key Laboratory of Cellular and Molecular Immunology, Key Laboratory of Educational Ministry of China, School of Basic Medical Sciences, Tianjin Medical University, Tianjin, China; ^4^ Department of Pediatrics, Division of Hematology/Oncology, Aflac Cancer and Blood Disorders Center, Emory University School of Medicine, GA, USA; ^5^ School of Pharmacal Sciences, Tianjin Medical University, Tianjin, China; ^6^ Department of Genetics, College of Basic Medicine, Tianjin Medical University, Tianjin, China

**Keywords:** circular RNAs, cZNF292, glioma, tube formation, proliferation

## Abstract

CircRNA is a novel type of RNA molecule formed by a covalently closed loop which have no 5′-3′ polarity and possess no polyA tail and relatively stable due to the cyclic structure. Therefore, they may serve as potential targets and diagnosis biomarkers for tumor therapy. cZNF292 is an important circular oncogenic RNA and plays a critical role in the progression of tube formation. This study is aimed at exploring the role of cZNF292 in human glioma tube formation and its potential mechanism of action. We found that cZNF292 silencing suppresses tube formation by inhibiting glioma cell proliferation and cell cycle progression. Cell cycle progression in human glioma U87MG and U251 cells was halted at S/G2/M phase via the Wnt/β-catenin signaling pathway and related genes such as PRR11, Cyclin A, p-CDK2, VEGFR-1/2, p-VEGFR-1/2 and EGFR. The results suggest that cZNF292 silencing plays an important role in the tube formation process and has potential for application as a therapeutic target and biomarker in glioma.

## INTRODUCTION

Gliomas are malignant tumors with the highest morbidity and mortality rate, and their highly aggressive and invasive nature contributes to poor prognoses in patients. In recent years, the prevalence of gliomas has increased [1–2]. Despite ongoing research, the mechanisms of gliomagenesis and glioma morbidity remain unclear. Exploring the molecular markers that regulate glioma morbidity and tumorigenesis would provide insight into glioma biological behaviors for potentially inhibiting disease progression.

CircRNA is a novel type of RNA molecule formed by a covalently closed loop and is found to exist widely in eukaryotes. CircRNAs are derived from gene exon or intron regions and are abundant in mammalian cells. Existing studies show that most circRNAs are conserved among different species [3–4]. Furthermore, circRNAs are relatively stable, owing to their cyclic structure and resultant resistance to degradation by RNase R. Due to their specific expression, complexity in regulation, and the important role they play in diseases, circRNAs are receiving increasing public attention [5]. Because circRNAs are closed loops, they have no 5′-3 ‘polarity and possess no polyA tail. Thus, they are more stable than linear RNAs and are unlikely to be degraded by RNA exonucleases or RNase R. As such, they may serve as potential targets for tumor therapy or diagnosis biomarkers, and they warrant further investigation [6].

cZNF292 is a circRNA found to be expressed during hypoxic conditions and cZNF292 silencing suppreses tube formation *in vitro*, suggesting that it may be related to the morbidity, development and prognosis of tumors [7], at same time, we found that cZNF292 was also expression in gliomas cell lines such as U87MG and U251. In this study, we hypothesized that cZNF292 plays an important role in gliomas tube formation, and we explored the role of cZNF292 on gliomas cell proliferation and tube formation through gliomas U87MG and U251 cell lines *in vitro,* revealing its possible underlying mechanisms of action.

## RESULTS

### cZNF292 silencing suppresses the proliferative and angiogenic potential of glioma cells *in vitro*

**Figure 1:**
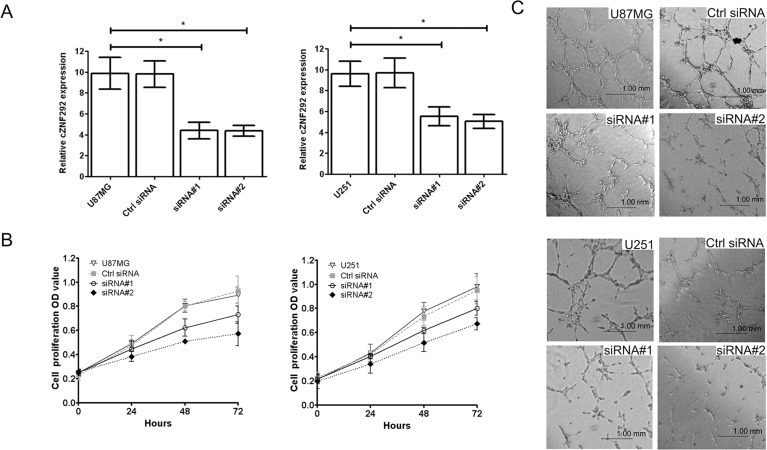
Effect of cZNF292 on the potential of proliferation and tube formation of glioma cells *in vitro* (**A**) The effects of the cZNF292 siRNA plasmid on the expression of cZNF292 in glioma U87MG and U251 cell lines. (**B**) The effect of cZNF292 silencing on the proliferation potential of the glioma U87MG and U251 cell lines. (**C**) The effect of cZNF292 silencing on tube formation in the glioma U87MG and U251 cell lines. (× 200) Error bars, ± SD; *P < 0.05.

To investigate the role of cZNF292 in the regulation of proliferation and tube formation, we silenced the expression of cZNF292 in the U87MG and U251 cell lines by cZNF292 siRNAs plasmids (Figure 1A), however, there was not a changed expression of linear transcript of cZNF292 (ZNF292) (not showed in figure). We found that cZNF292 silencing suppressed proliferation (Figure 1B) and tube formation (Figure 1C).

**Figure 2:**
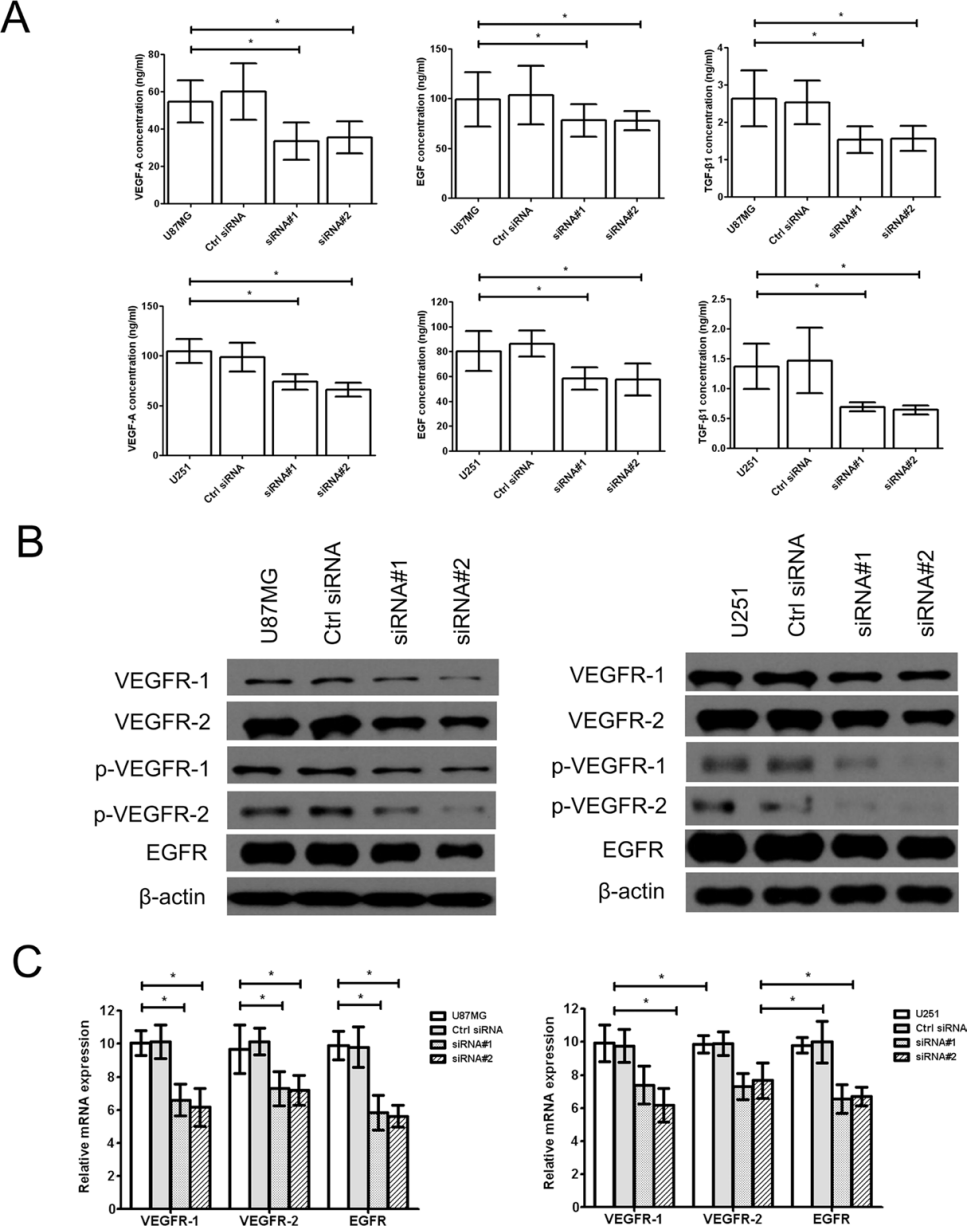
Effect of cZNF292 on the expression of tube formation related gene of glioma cells *in vitro* (**A**) The effects of cZNF292 silencing on the content of VEGF-A, EGF and TGF-β1 in the supernatants from the tube formation assays performed using the glioma U87MG and U251 cell lines. (**B**) The effects of cZNF292 silencing on the protein expression of VEGFR-1/2, p-VEGFR-1/2 and EGFR in the glioma U87MG and U251 cell lines. (**C**) The effects of cZNF292 silencing on the mRNA expression of VEGFR-1/2 and EGFR in the glioma U87MG and U251 cell lines. Error bars, ± SD; *P < 0.05.

Additionally, the vascular endothelial growth factor A (VEGF-A) and its receptor VEGFR-1/2 were interacted to affect to tube formation of tumor cells by TGF-β1, at same time, EGF/EGFR also has similar activity. Therefore, we found that the levels of VEGF-A, EGF and active TGF-β1 (Figure 2A) and the expression of vascular endothelial growth factor receptor-1/2 (VEGFR-1/2), phosphorylated vascular endothelial growth factor receptor-1/2 (p-VEGFR-1/2) and epidermal growth factor receptor (EGFR) (Figure 2B, 2C) were significantly downregulated in the cZNF292-silenced U87MG and U251 cell lines, suggesting that cZNF292 silencing represses tumor proliferation and tube formation in glioma cells.

### cZNF292 silencing blocks glioma cell cycle progression by Wnt/β-catenin signaling pathway *in vitro*

**Figure 3:**
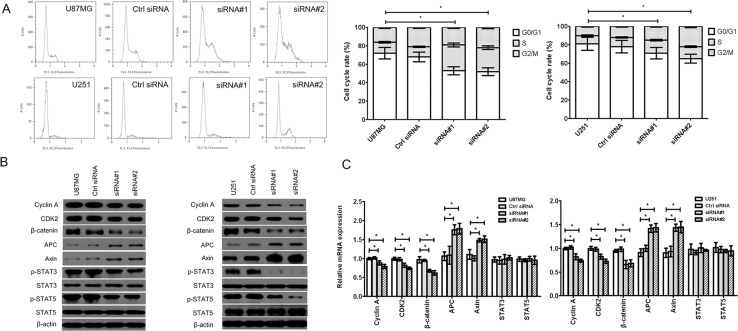
Effect of cZNF292 on the cell cycle of glioma cells *in vitro* (**A**) cZNF292 silencing arrested the cell cycle at S/G2/M phase in the glioma U87MG and U251 cell lines. (**B**) The effects of cZNF292 silencing on the protein expression of Cyclin A, CDK2, p-CDK2, β-catenin, APC, Axin, p-STAT3, p-STAT5, STAT3, STAT5 and PRR11 in the glioma U87MG and U251 cell lines. (**C**) The effects of cZNF292 silencing on the mRNA expression of Cyclin A, CDK2, β-catenin, APC, Axin, STAT3, STAT5 and PRR11 in the glioma U87MG and U251 cell lines. Error bars, ± SD; *P < 0.05.

Cell cycle progression could be regulated by Wnt/β-catenin signaling pathway (β-catenin, APC, Axin, STAT3 and STAT5 were the key involvements) and its downstream molecule, such as Cyclin A, CDK2 and PRR11. We found that the cell cycle was arrested in S/G2/M phase (Figure 3A) in the cZNF292-silenced U87MG and U251 cell lines. The expression of Cyclin A, CDK2,p-CDK2, β-catenin, p-STAT3 (Tyr705), p-STAT5 (Tyr694) and proline-rich protein 11 (PRR11) was downregulated, the expression of Axin and adenomatous polyposis coli (APC) was upregulated; and the expression of STAT3 and STAT5 was not significantly change (Figure 3B, 3C), suggesting that cZNF292 silencing represses tumor tube formation through regulating cell cycle by Wnt/β-catenin signaling pathway.

### cZNF292 silencing represses the activity of glioma cell transcription factors *in vitro*

**Figure 4:**
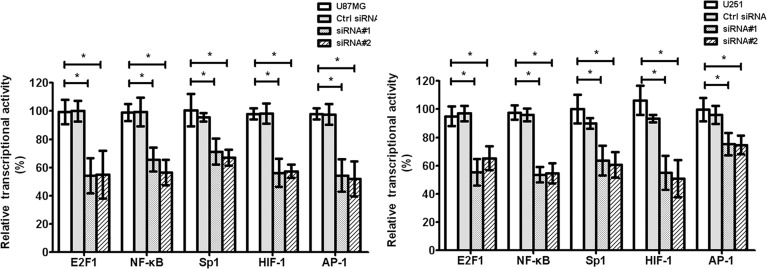
Effect of cZNF292 on the activity of transcriptional factors of glioma cells *in vitro* The effect of HULC silencing on the transcriptional activity of E2F1, NF-κB, Sp1, HIF-1 and AP-1 in the glioma U87MG and U251 cell lines. Error bars, ± SD; *P < 0.05.

The transcription factors, such as E2F1, NF-κB, Sp1, HIF-1, AP-1, STAT3, and STAT5, play important roles in tumor tube formation. We found that cZNF292 silencing decreased the transcriptional activity of E2F1, NF-κB, Sp1, HIF-1, AP-1, STAT3, and STAT5 in the U87MG and U251 cell lines (Figure 4), suggesting that cZNF292 silencing represses tumor tube formation through altering transcription factor activity.

## DISCUSSION

CircRNAs were originally considered to be a byproduct of RNA transcription and splicing and their expressive abundance is low; therefore, historically, they have not been regarded as biologically important molecules [8]. Studies have shown that the formation of circRNAs takes place by backsplicing, a different mechanism from the standard splicing mode of linear RNA. Additionally, they may form by the combination of endogenous miRNAs. Some circRNAs with intron retention are positioned in the eukaryotic nuclei, where they may regulate gene expression, suggesting their potential use as therapeutic targets or biomarkers for disease diagnosis [9–10].

One study has found that cZNF292 can be upregulated in hypoxic conditions, such as those observed during human umbilical vein endothelial cell (HUVEC) tube formation, and this same study confirmed the role of cZNF292 in tumorigenesis. Similarly, it is herein reported that siRNA knockdown of cZNF292 expression significantly inhibits proliferation and tube formation of U87MG and U251 cells.

Cyclin A is a key molecular regulator of cell cycle progression from S to G2 phase, which works through activating CDK2. This regulatory pathway is involved in cell proliferation and tube formation and is closely associated with tumor development and morbidity [11–12]. Additionally, recent studies have shown that proline-rich protein 11 (PRR11), which is also involved in cell cycle regulation, is related to tumor malignancy [13]. Our results show that cell cycle regulation was arrested in S/G2/M phase by cZNF292 silenced may be a potential mechanism underlying the effect of cZNF292 on tube formation.

EGFR, VEGF-A, and the VEGF-A receptor VEGFR-1/2 are believed to play key regulatory roles in tumor tube formation [14–16]. This study found that the expressions of VEGFR-1/2, p-VEGFR-1/2, and EGFR are downregulated after cZNF292 silencing, suggesting that these genes are involved in glioma cZNF292 biology.

Axin, β-catenin, Adenomatous polyposis coli (APC) and STAT3/5 is the integral component of the Wnt/β-catenin signaling pathway which plays a critical role in cell growth, differentiation and cancer process. Axin is known to be the tumor suppressor gene was associated with the other colorectal tumour suppressor APC, their central function of axin is to degradation of β-catenin, which is a downstream signaling molecule of Wnt/β-catenin signaling pathway, which regulates many biological functions in tumor cells, to prevent the activation of Wnt/β-catenin signaling pathway [17].

The transcription factors E2F1, NF-κB, Sp1, HIF-1, AP-1, STAT3, and STAT5 play important roles in tumor cell signal transduction [18–21]. We found that cZNF292 silencing can significantly downregulate the activity of the above transcription factors and the expression of β-catenin, suggesting that signal transduction in the STAT3/5/β-catenin pathway is also a potential mechanism through which cZNF292 exerts its regulatory role in glioma cells.

In conclusion, this study shows that cZNF292 plays an important role in glioma proliferation and tube formation. The mechanism of cZNF292 activity may involve the regulation of the cell cycle and related genes. However, more in-depth mechanistic studies are needed to further understand the mechanism of cZNF292 activity.

## MATERIALS AND METHODS

### Cell culture and transfection

The human glioma U87MG and U251 cell lines were obtained from the American Type Culture Collection (USA) and were cultured in standard conditions (DMEM with 10% fetal bovine serum (FBS) (Gibco) at 37°C with 5% CO_2_).

Cells were cultured in a 6-well plate for 24 h and subsequently transfected with cZNF292 siRNA plasmids (50 nM) using GeneJuice (Merck), according to the manufacturer's protocol for 6 h. Then, fresh culture medium was added and cells were harvested after 72 h in all experiments. siRNA synthesis and construction of pcDNA3.1 plasmids containing cZNF292 siRNAs (#1: 3′-CAGAACACACACUAUAGAG-5′l; #2: 3′-AUCUAGUAGCUCCCUUAAA-5′) or a control scrambled siRNA (3′-UCUCUCACAACGGGCAUUU-5′, Ctrl siRNA) were conducted by our laboratory.

### Cell proliferation assay

Five thousand cells were seeded into each well of a 96-well plate and cultured in standard conditions for 72 h. Then, a BrdU cell proliferation kit (Abcam) was used to determine the optical density (OD), according to the manufacturer's instructions, using a microplate reader (Thermo Scientific Multiskan FC) at 450 nm every 24 h.

### Tube formation assay

Matrigel (BD Biosciences) was added to each well of a 24-well plate and stored overnight at 4°C. Then, 5 × 10^4^ cells were seeded, incubated at 37°C overnight, and stained with Calcin at 37°C for 30 min. Tube formation was observed with a microscope (× 200) after washing with PBS.

### Cell cycle assay

Cells (3 × 10^5^) were seeded into each well of a 6-well plate and cultured in standard conditions for 72 h. Then, the cells were harvested, fixed with 70% cold ethanol at 4°C overnight and washed with PBS. The cells were then stained with propidium iodide (PI) with RNaseA (100 μg/mL) for 30 min in the dark and washed with PBS, and the cell cycle distribution was assessed using a FACSAria flow cytometer (BD Biosciences) at 488 nm.

### Liquid-chip assay

The levels VEGF, EGF, and TGF-β1 in the supernatants from the tube formation assay were measured using a liquid-chip assay according to the manufacturer's instructions (R & D Systems). The concentration of each cytokine was determined using a standard curve according to the kit's instructions.

### Western blot

Cells (3 × 10^5^) were seeded into each well of a 6-well plate, cultured in standard conditions for 72 h, collected, lysed with RIPA lysis buffer (Millipore), and pelleted by centrifugation at 12,000 g at 4°C for 10 min. Total protein concentrations were measured with a bicinchoninic acid (BCA) kit (Sigma Aldrich), and 100 μg protein samples were separated in a 12% SDS-PAGE gel and transferred to a PVDF membrane through 10% SDS-PAGE electrophoresis (150 mA, 1 h). After the membrane was blocked with 1% BSA at room temperature for 1 h, the protein expressions of β-catenin, p-STAT3 (Tyr705), p-STAT5 (Tyr 694/Tyr 699), STAT3, STAT5, PRR11, Cyclin A, CDK2, p-CDK2, APC, Axin, VEGFR-1/2, p-VEGFR-1/2 and EGFR were detected through incubation overnight at 4°C with primary antibodies (Supplementary Table S1). The membrane was washed with PBST (Phosphate Buffer Solution with 0.05% Tween20) and incubated with peroxidase-labeled anti-rabbit IgG at room temperature for 1 h. An ECL kit (Millipore) was used to show the immunoreactive band after the membrane was washed with PBST. β-actin was used as the internal reference.

### Real-time PCR assay

Cells (3 × 10^5^) were seeded into each well of a 6-well plate and cultured in standard conditions for 72 h. RNA was isolated using Qiazol (Qiagen) according to the manufacturer's protocol. SYBR Green I dye was used for reverse transcription in an ABI 7500 fluorescence quantitative PCR instrument, and the mRNA levels of β-catenin, STAT3, STAT5, PRR11, Cyclin A, CDK2, APC, Axin, VEGFR-1/2 and EGFR were measured (Supplementary Table S2). The PCR condition was as follows: denaturation at 95°C for 10 min, followed by 40 cycles at 95°C for 15 s, 60°C for 60 s and a final elongation at 95°C for 15 s, 60°C for 60 s and 95°C for 15 s. β-actin was used as the internal reference, and the 2^−ΔCt^ method was used for calculating mRNA expression ratios.

### Reporter gene assay

Inducible luciferase plasmids (pcDNA3.1) carrying the genes for E2F1, NF-κB, Sp1, HIF-1, AP-1, STAT3, and STAT5 were constructed by our laboratory, and a constitutively expressed Renilla-luciferase pcDNA3.1 plasmid (Qiagen Co. Ltd) was used as a control. Cells (3 × 10^5^) were seeded into each well of a 6-well plate and cultured in standard conditions for 24 h. The above-mentioned plasmids were transfected into the cells for 6 h using GeneJuice (Merck) according to the manufacturer's protocol. Then, fresh media was added, and the cells were incubated for another 72 h under standard conditions. The resultant luciferase activity was analyzed using the Bright-Glo™ Luciferase Assay System (Promega).

### Statistical analysis

The data are shown as the mean ± standard deviation ($\bar{x}\pm \text{s}$). One-way ANOVA analysis was performed using SPSS 10.0 software; significance is defined as *p* ≤ 0.05.

## SUPPLEMENTARY MATERIALS TABLES


