# Supplementary material for: Silencing of cZNF292 circular RNA suppresses human glioma tube formation via the Wnt/β-catenin signaling pathway
Source: Oncotarget. 2016 Aug 23;7(39):63449–55. doi: 10.18632/oncotarget.11523 (PMC5325376; doi:10.18632/oncotarget.11523)
Supplement: Supplementary file 1 [file oncotarget-07-63449-s001.pdf]

Silencing of cZNF292 circular RNA suppresses human glioma tube formation via the Wnt/β-catenin signaling pathway

Supplementary Materials

Supplementary Table S1: The anti-body used in the study

No.	gene	Manufacturer	Cat #	Ratio
1	p-STAT3	Santa Cruz Biotechnology	sc-7993	1:1200
2	p-STAT5 (Tyr 694/Tyr 699)	Santa Cruz Biotechnology	sc-11761	1:1200
3	STAT3	Santa Cruz Biotechnology	sc-293151	1:1500
4	STAT5	Santa Cruz Biotechnology	sc-74442	1:1500
5	β-catenin	Santa Cruz Biotechnology	sc-65480	1:2000
6	PRR11	Santa Cruz Biotechnology	sc-161167	1:1500
7	p-CDK2	Santa Cruz Biotechnology	sc-101656	1:1000
8	CDK2	Santa Cruz Biotechnology	sc-163	1:1500
9	CyclinA	Cell Signaling Technology	4656	1:1500
10	VEGFR-1	Cell Signaling Technology	2893	1:1500
11	VEGFR-2	Cell Signaling Technology	2479	1:1500
12	p-VEGFR-1	abcam	ab32152	1:1000
13	p-VEGFR-2	Cell Signaling Technology	2478	1:1000
14	β-actin	Sigma Aldrich	A228	1:5000
15	Anti-rabbit IgG, HRP-linked Antibody	Cell Signaling Technology	7074	1:2000
16	Anti-mouse IgG, HRP-linked Antibody	Cell Signaling Technology	7076	1:2000
17	Anti-Goat IgG, HRP-linked Antibody	Sigma Aldrich	A5420	1:2000

Supplementary Table S2: The primers used in the study

Gene	Primer Sequences (5′-3′)
cZNF292	Forward: GCTCAAGAGACTGGGGTGTG<br>Reverse: AGTGTGTGTTCTGGGGCAAG
STAT3	Forward: ACCAGCAGTATAGCCGCTTC<br>Reverse: GCCACAATCCGGGCAATCT
STAT5	Forward: ACATTTGAGGAGCTGCGACT<br>Reverse: CCTCCAGAGACACCTGCTTC
β-catenin	Forward: CAGCAGCAATTTGTGGAGGG<br>Reverse: GCAGCTGCACAAACAATGGA
PRR11	Forward: CGTATCTGCCACCGGAACTT<br>Reverse: GAGATGGTCTTCAGTGCTTCCT
CDK2	Forward: GCCAGAAACAAGTTGACGGGAGA<br>Reverse: TGGGTGTAAGTACGAACAGGGAC
CyclinA	Forward: GCCATTAGTTTACCTGGACCCAGA<br>Reverse: CACTGACATGGAAGACAGGAACCT
TGF-β1	Forward: GACTACTACGCCAAGGAG<br>Reverse: TGAGGTATCGCCAGGAAT
EGF	Forward: AGCCAGCTCTGATCTAATCTGG<br>Reverse: TTTTGCAAATATGTTCACAGCC
EGFR	Forward: GTCTCTTGCCGGAATGTCAG<br>Reverse: CTCACCCTCCAGAAGGTTGC
VEGF-A	Forward: CTACCTCCACCATGCCAAGT<br>Reverse: GCAGTAGCTGCGCTGATAGA
VEGFR-1	Forward: CGGAAGGAAGACAGCTCATC<br>Reverse: CTTCACGCGACAGGTGTAGA
VEGFR-2	Forward: GCTTTCGGTAGTGGGATGAA<br>Reverse: GGAATCCATAGGCGAGATCA
β-actin	Forward: AGGCACCAGGGCGTGAT<br>Reverse: GCCCACATAGGAATCCTTCTGAC
